# Hybrid Deep Learning Techniques for Securing Bioluminescent Interfaces in Internet of Bio Nano Things

**DOI:** 10.3390/s23218972

**Published:** 2023-11-04

**Authors:** Taimur Bakhshi, Sidra Zafar

**Affiliations:** 1 School of Built Environment, Engineering & Computing, Leeds Beckett University, Leeds LS1 3HE, UK; 2 Department of Computer Science, Kinnaird College for Women, Lahore 54000, Pakistan; sidra.zafar@kinnaird.edu.pk

**Keywords:** deep learning, internet of bio-nano things, bioluminescence, bio–cyber interface, intrusion detection, anomaly detection, traffic classification, neuromorphic computing

## Abstract

The Internet of bio-nano things (IoBNT) is an emerging paradigm employing nanoscale (~1–100 nm) biological transceivers to collect in vivo signaling information from the human body and communicate it to healthcare providers over the Internet. Bio-nano-things (BNT) offer external actuation of in-body molecular communication (MC) for targeted drug delivery to otherwise inaccessible parts of the human tissue. BNTs are inter-connected using chemical diffusion channels, forming an in vivo bio-nano network, connected to an external ex vivo environment such as the Internet using bio-cyber interfaces. Bio-luminescent bio-cyber interfacing (BBI) has proven to be promising in realizing IoBNT systems due to their non-obtrusive and low-cost implementation. BBI security, however, is a key concern during practical implementation since Internet connectivity exposes the interfaces to external threat vectors, and accurate classification of anomalous BBI traffic patterns is required to offer mitigation. However, parameter complexity and underlying intricate correlations among BBI traffic characteristics limit the use of existing machine-learning (ML) based anomaly detection methods typically requiring hand-crafted feature designing. To this end, the present work investigates the employment of deep learning (DL) algorithms allowing dynamic and scalable feature engineering to discriminate between normal and anomalous BBI traffic. During extensive validation using singular and multi-dimensional models on the generated dataset, our hybrid convolutional and recurrent ensemble (CNN + LSTM) reported an accuracy of approximately ~93.51% over other deep and shallow structures. Furthermore, employing a hybrid DL network allowed automated extraction of normal as well as temporal features in BBI data, eliminating manual selection and crafting of input features for accurate prediction. Finally, we recommend deployment primitives of the extracted optimal classifier in conventional intrusion detection systems as well as evolving non-Von Neumann architectures for real-time anomaly detection.

## 1. Introduction

The Internet of Things (IoT) presents a connected environment where real-life objects like sensors, actuators, and electronic devices can interact with each other through enabling technologies such as the Internet. The concept of IoT has recently been revised in the light of novel nanotechnology tools and synthetic biology, which have resulted in the fabrication of biologically embedded computing devices at the scale of nanometers (1–100 nm) called Bio-Nano things (BNT) [1]. The minute size puts constraints on their operational resources like computational complexity and storage capabilities. Therefore, bio-nano things are only able to perform trivial tasks like sensing, data storage, and actuation. The capabilities of a single BNT device, in terms of complexity and range of operation, can be expanded when allowed to interact with counterparts forming a network called bio-nanonetwork (BNN). Nanonetwork enables bio-nano things to share, fuse, and coordinate their information in the biological environment through a novel communication technology called Molecular Communication (MC) [2]. MC enables the biochemical processing of data, the transformation of chemical energy, and the exchange of information through the transmission and reception of molecules. These characteristics of MC can be advantageous for environments like the human body, where the use of electromagnetic (EM) radiation for healthcare could result in unwanted effects on human tissue. The connection of MC to external networks such as the Internet leads to a novel communication paradigm called the Internet of Bio-Nano Things (IoBNT) [1,3]. IoBNT opens up a plethora of biomedical applications such as real-time intra-body sensing and actuation, targeted drug delivery (TDD), and tissue re-engineering [4]. IoBNT applications, allow TDD and remote patient monitoring, in the ease of patients’ home or workplace by the healthcare provider. It can, therefore, be inferred that IoBNT interfaces will aid in connecting the biological environment of the human body to the electrical cyber world. In this scenario, where the human body can be monitored and injected with drugs remotely, the security of the users is of paramount importance. Several cyber-attacks and viruses can be launched on the bio-cyber interface to disrupt in vivo MC-related information being sent to the healthcare provider, as well as alter or disrupt physiological functions and homeostasis of the body, giving birth to a new form of terrorism called *bio-cyber terrorism* [1,4]. Among the existing proposals for bio-cyber interfacing technologies, bioluminescent-based interfaces (BBI) have demonstrated realistic incorporation in the IoBNT paradigm due to their low cost and highly efficient operation [4,5]. Securing BBI against inherent threat vectors requires state-of-the-art intrusion detection systems (IDS). This work proposes a comprehensive anomaly detection framework for bioluminescence-based bio–cyber interface (BBI) in IoBNT systems using deep learning classification. A combination of Deep Learning-Convolutional Neural Network (CNN) and Long Short-Term Memory (LSTM) based profiling scheme is used to differentiate the anomalous activity of bio–cyber interface. In the case of traffic classification, CNN alone cannot perform well as it does not specifically account for temporal characteristics. In a malicious attack, several traffic modalities can be altered in a short period of time to modify in-vivo bio-interface parameters. In this situation applying CNN alone may lead to missed alerts. Therefore, CNN is combined with LSTMs, which support sequence prediction and account for time-related characteristics of abnormal operations. The hybrid CNN-LSTM architecture involves using CNN layers for feature extraction on input data and LSTM to analyze sequence prediction and temporal information. The proposed security framework is trained using extensive simulations on data presenting significant attributes of bioluminescence-based bio–cyber interface. We address the system engineering perspective by proposing and comparing different scenarios for implementing deep learning (DL) classifiers for anomaly detection in IoBNT systems. The rest of this paper is organized as follows. Section 2 highlights the background on the Internet of Bio Nano things and bio–cyber interfacing, presents related work, and presents applications of deep learning in cyber security. Section 3 discusses bio–cyber interfacing, and measurement parameters to be used for anomaly detection. Section 4 discusses the proposed methodology, data collection and pre-processing, and the neural network structures used. Section 5 evaluates the results from a qualitative and quantitative perspective and presents system deployment scenarios for the derived classifier. Final conclusions are drawn in Section 6.

## 2. Background

In the present section Internet of Bio Nano Things, related work, and an overview of deep learning techniques in network security are summarized.

### 2.1. Internet of Bio Nano Things-Overview

**Figure 1:**
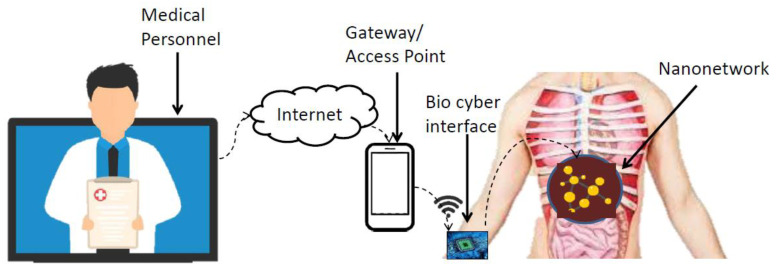
IoBNT architecture: command-feedback signal transceiver using bio–cyber interface over the Internet.

The architecture of IoBNT is a holistic framework that encapsulates heterogeneous devices (nano-macro scale) and communication protocols (nano communication to macro-scale communication). A reference architecture of IoBNT is presented in Figure 1. The IoBNT paradigm was first proposed by Akyildiz et al. [1] and followed by further research contributions in the design and technological aspects of IoBNT such as bio–cyber interfacing by Chude-Okonkwo et al. [5] and Nakano et al. [2], and molecular communication primitives by Nakano et al. [6,7] and and Felicetti et al. [8]; nanonetworks by Akyildiz et al. [3]; communication channel characteristics by Garralda et al. [9], Kuran et al. [10], and Gregori and Akyildiz [11]. The basic unit of IoBNT is the Bio-Nano Thing (BNT). Bio-Nano things are often referred to as nano things, nanomachines, and nano devices in literature. The size of bio nano things is of the order of nanometres (100 nm). Bio-Nano things can be composed of only biological materials (lipids, proteins, etc.) or can be coated with non-biological materials like magnetic particles. The design of bio-nano things can be customized according to the task at hand, e.g., liposomes are capable of storing and releasing molecules, long circulation, targeting, and stimuli responsiveness [12,13]. Genetically engineered biological cells, bacterium, and artificial cells have the capabilities to synthesize and emit specific molecules and thus can also be used as bio-nano things [5]. To equip the bio-things with sensing abilities, a special nanosensor can be designed using a whole cell [14,15] and sensor molecule entrapped in a chemically inert matrix [16]. All the above-mentioned bio-nano things collaboratively work in the nano-networking setup. Bio-nano things traverse the blood vessel network and transport the encoded molecular information to the bio–cyber interface. The design of Bio–cyber interface is an open research issue in the realization of the IoBNT paradigm. Research efforts have been devoted towards interfacing technologies such as electronic tattoos, hydro-gel conductors, and chemo-electro transduction units to offer connectivity between the human body and the Internet with considerable success [5,17,18,19]. Among the existing interfacing technologies bioluminescent interfacing has provided promising results in practical implementation owing economy of cost and ease of deployment [4,5,7,9].

### 2.2. Related Work

The realization of a secure bio–cyber interface will aid in wider adoption of the promising healthcare applications of IoBNT. Bioluminescence-based IoBNT is moving towards greater realization, requiring security primitives able to monitor and identify in vivo traffic anomalies triggered by external (Internet) cyber-attacks [4]. A brief overview of the state-of-the-art IoBNT security and complementary research in associated domains is presented in Table 1. Pioneering surveys such as [1,2,4,6] have primarily focused on systematically reviewing bio–cyber interfacing technologies and documenting underlying security requirements. Research contributions in formulating a holistic intrusion detection and prevention (IDS, IPS) framework for IoBNT are, however, still nascent. In the direction of molecular communication (MC), some preliminary studies have been conducted to explore the security requirements of nanonetworks, attack categorization, as well as authentication mechanisms [20,21]. Giaretta et al. [22] classified different attack types (black hole and sentry) along with their countermeasures for BNT security. Zafar et al. [23] proposed an authentication scheme for diffusion-based MC using Channel Impulse Response (CIR) as a device fingerprint. Recent studies have sought to use machine learning-based approaches to classify and discriminate network traffic for bio–cyber interface security. El-Fatyany et al. [24] presented an authentication scheme for bio luminescence-based bio–cyber interface (BBI). The proposed chaotic model utilizes a modified logistic map to control drug release in a targeted drug delivery system. Similarly, an earlier work, by Bakhshi et al. [25] devised an authentication scheme for bio–cyber interface technologies using ML-enabled profiling. The security framework was designed for three types of bio–cyber interfacing technologies namely bioFET, bioluminescence, and reduction-oxidation (redox) modality. However, the study focused on using only a limited number of external traffic parameters as input features for ML training. While ML-based profiling utilizing manually crafted input parameters have proven to be somewhat efficient in traditional IDS (and IPS), the inter-dependence and inherent complexity of bio-nano traffic constrains their optimality for anomaly detection in the IoBNT domain. Increasing levels of data (traffic) intricacy and a requirement for greater classification accuracy have led researchers to investigate applications of deep learning neural networks in the mainstream IoT domain. DL network structures have been shown to aid dynamic feature selection and significantly reduce false positives during traffic classification in heterogeneous IoT settings [26,27]. Abusitta et al. [26] highlighted a preference for DL-based classification to recognize abnormal behavior in IoT traffic, prone to external noise over common ML algorithms. Using de-noising autoencoders during the data pre-processing stage, the DL classifier was able to discriminate malicious traffic patterns by dynamically extracting useful features despite an unstable operational environment. Similarly, Rosero-Montalvo et al. [27] used a hybrid DL ensemble to detect anomalies with minimal memory and computational footprint in high-noise IoT environments. Real estate and workload economy enabled DL classifier realization on off-shelf IoT sensory hardware as well as ensured high bandwidth efficiency. Chander et al. [28] focused on industrial IoT (IIoT) sensors, developing metaheuristic feature selection, and tested single and multidimensional cascaded recurrent neural networks for the identification of anomalies. The resulting classifier allowed satisfactory anomaly detection accuracy which was further improved by model tuning. Hameed et al. [29] compared the efficacy of existing ML solutions for IoT traffic classification in smart cities. The authors were of the view that fine-grained flow characteristics and correlations among IoT traffic parameters presented a computational concern in typical ML-based classifiers. Using multi-layer perceptron—artificial neural networks (MLP-ANN), and a two-stage learning framework researchers were able to achieve high accuracy and precision in identifying IoT devices based on network traffic, significantly outperforming well-known ML algorithms. Guan J. et al. [30] highlight the scarcity of IoT labeled data in 5G environments, the limited capability of devices, and the significant efforts involved in artificially designing features for traffic classification. Employing deep transfer learning, the researchers used customized convolutional networks trained on resource-constrained targets to provide strong traffic classification accuracy. Reducing human intervention during feature selection, the framework decreased system complexity for future adoption in 5G IoT networks to isolate traffic of interest. Etemadi et al. [31] surveyed abnormality detection at the molecular communication level and compared different ML, heuristics, and DL primitives for this purpose. The work discussed the viability of ML approaches, as well as the utilization of naturally inspired systems based on genetic, particle swarm, and colony optimization algorithms for identifying anomalous (traffic) activity. It was observed that while these techniques are appropriate for stationary sensors and objects, it became very challenging to address moving sensors and abnormality sources. The authors recommended using a combination of heuristic deep learning methods and localized customization to identify anomalous behavior in IoBNT. The application of DL techniques for anomaly detection in network engineering, however, remains prevalent [32,33]. Preliminary efforts in using DL-based anomaly detectors for bioFETs have been investigated by Zafar et al. [33]. The authors proposed an Artificial Neural Network-based classifier for biological field effect transistor (bioFET) based bio–cyber interface. Particle Swarm Optimization (PSO) was used to adjust the weights and biases of the neural networks to provide better classification. The proposed model utilizes communication and design parameters of bioFET-based bio–cyber interface to classify normal versus abnormal traffic behavior. The present work seeks to expand on the utility of DL-based network structures through parameter profiling of bioluminescent interfaces and allowing timely detection of anomalies to realize secure avenues for IoBNT deployment in future healthcare applications. 

### 2.3. Deep-Learning Enabled Traffic Analysis

Traditional machine learning techniques including Iterative Dichotomiser 3 (ID3), decision tree (C 5.0), classification and regression tree (CART), and artificial neural network (ANN) have been previously recommended to provide a measure of security for IoBNT biocyber interfaces [25,26]. Deep learning is a sub-field of traditional machine learning, providing increased flexibility and accuracy over classical learning algorithms, and offering the ability to incrementally learn and extrapolate new features from a limited set of training data [34,35]. Moreover, the thin and layered structure of sequential deep neural network models makes them ideal for being deployed over a low-powered and resource-constrained bio–cyber interface, still facilitating real-time anomaly detection. Two of the popular deep learning network types being used in several image recognition, video editing, industrial, and security systems include the convolutional and recurrent neural networks [34].

Convolutional neural network (CNN) is an extension of the traditional feed-forward neural network. CNN has been proven significantlyly successful in image processing tasks including object identification in robotics, and self-driven cars, and are now gaining traction in cyber security application particularly anomaly detection [32,34,35,36]. Four main operations of CNN comprise of a convolution layer responsible for feature extraction, a non-linear activation function such as Rectified Linear Unit (ReLu) for fast(er) model learning, a pooling layer for generalization, and a fully connected layer (to be used for classification) [37]. The number of neurons in hidden layers relates to model learning ability, albeit at the cost of computation. CNNs are extremely useful in image processing applications, however, usually offer limited capability in effectively interpreting temporal data characteristics inherent in cyber security primitives, traffic classification, and intrusion detection systems [36]. 

Recurrent neural networks (RNN) structures are used for processing time-series data by employing neurons capable of remembering previously processed (data) states [38]. RNNs are based on human-cognitive functionality and can generate input-output mappings considering time-dimensionality (i.e., order of occurrence). The network structure is based on feedback loops, using the previous state to compute the next state. For longer data sequences, RNNs however, suffer from a vanishing gradient problem (short-term memory) resulting in high latency and signal transfer deviation from one (prior) iteration to the next. To deal with vanishing gradient and to address memory problems, variations in RNN including the long-short term memory (LSTM) and gated recurrent unit (GRU) have been proposed [39].

Long short-term memory (LSTM) is a refined extension of RNNs, developed to reduce the gradient explosion and dispersion issue. LSTM introduces memory blocks, in contrast to the conventional simple RNN units. A memory block contains a memory cell and a set of gates i.e., input, forget, and output. This has emerged to be more effective in sequence information analysis and capturing long-range dependencies with applications in network security [35]. LSTM filters prior states based on greater influence on the present. 

Gated recurrent unit (GRU) is also a type of RNN comprising of an update and reset gate, trained to select, and retain information that needs to be fed to the output. The update gate allows the determination of past information to be sent to future states fully or partially and significantly reduces the vanishing gradient problem. The reset gate determines the scope of forgetting past information. While the applicability of CNN and RNN structures is widespread in image processing and handwriting recognition, their applicability in anomaly detection systems is relatively nascent [4,24]. To the best of our knowledge, the present work is the first to consider deep learning structures for the security of IoBNT devices and interfaces.

## 3. Bioluminescence Based Bio–Cyber Interface Architecture

**Figure 2:**
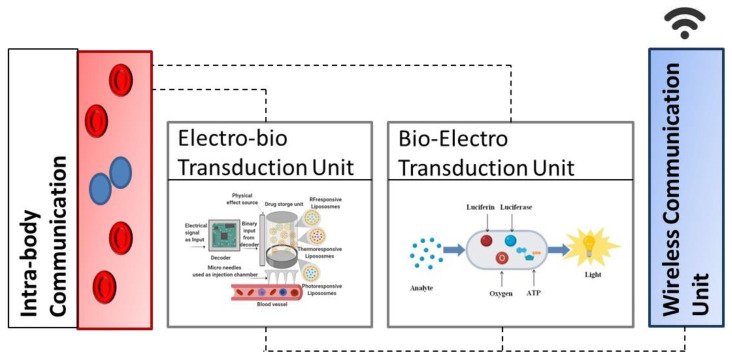
An illustration of a generic bio–cyber interface.

Bio–cyber interface is a hybrid device capable of transducing in-body biochemical signals into electromagnetic signals and vice-versa. Several modalities can be used for transduction between biochemical and electromagnetic signals such as bioFET-based molecular antenna [17], reduction-oxidation (Redox) modality [18,19], and other interfaces offering biologically inspired capabilities like thermo-responsiveness and photo-responsiveness [5]. The choice of interfacing modality depends upon the assigned application requirements and propagation channel [4]. In this work, we have considered the bioluminescence-based biocyber interface model proposed by Chude-Okonkwo et al. [5] based on biologically inspired properties such as biocompatibility, biodegradability, and non-toxicity. The bioluminescent biocyber interface (BBI) provides a practical opportunity for healthcare professionals to connect the Internet to in-body (in vivo) bio nanonetworks and studies in the domain are maturing to prototype evaluations [40]. We present a sample architecture and bio-cyber interface model illustrated in Figure 2. The bio-cyber interface utilizes thermal and light responsiveness to certain biomolecules producing information signals (biochemical reactions) propagated using the blood vessel network to an in vivo nanonetwork location for targeted drug delivery. The architecture of the bio–cyber interface includes two units: an electro-bio transduction unit and a bio-electro transduction unit. A detailed description of these units is given below.

### 3.1. Electro-Bio Transduction Unit

**Figure 3:**
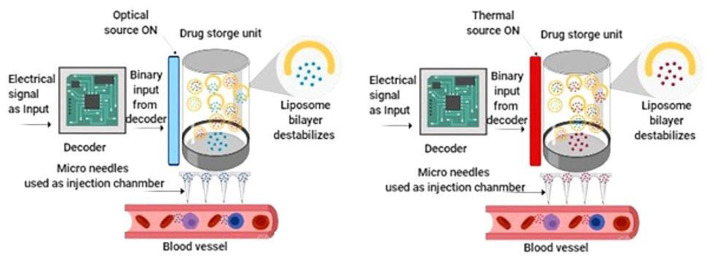
Electro-bio transduction unit on left when the optical source is ON and on the right when the thermal source is ON.

Electro-bio transduction unit (EBTU) converts electrical signals/commands received from medical personnel into biochemical signals. A visual illustration of EBTU is provided in Figure 3. 

The command received from external devices is in the form of a binary code. This binary code is used to drive logic gates to produce an optical or thermal effect. The unit also contains a small chamber/drug reservoir that contains nano-sized (1–100 nm) carriers. These nanocarriers are designed in such a way that they release their contents upon stimulation from external factors such as changes in temperature, light, pH, etc. The bio–cyber interface model based on [5] adopted for this work employs two types of liposomes: *photo-responsive* liposomes and *thermo-responsive* liposomes. The photo-responsive release is considered through *photoisomerization*, where liposomes encapsulate molecules that excite upon the light illumination from an external source, this causes a conformational change and destabilization of the lipid membrane that allows molecule release. For thermo-responsiveness, molecules are encapsulated in temperature-sensitive liposomes or dendrimers [41], which deteriorate upon receiving nonlinear sharp changes in the temperature. To preserve the contents during propagation, thermo-responsive liposomes must retain their load at body temperature (37 °C) and may release their contents within a locally heated microenvironment. The release process of liposomes can be expressed in the following equation:

v(t) = ε_T_ (1 − e^(γt)^) (1)

where ε_T_ is the cumulative molecular concentration and γ is the release rate of liposomes. The output of the electro-bio unit can be expressed as follows.

c^(f)^ = ∫ _(0→Tin)_ ε Ψ (t) dt (2)

where T_in_ is the time difference between injection and the start of the release process and Ψ is the total number of liposomes that release their content. The communication path of the electro-bio transduction unit is from bio–cyber interface towards in-body nanonetworks. The concentration of molecules may change during the propagation time i.e., after being injected from the bio–cyber interface into the blood vessel network and reaching the nano network or designated tissue. This rate of change of molecular communication from electro-bio transduction unit towards nanonetwork can be modeled through the equation below [5]:

d_t_v_1_(t) = v_1_(t) (k_12_ + k_10_) + k_21_ v_2_(t) (3)

d_t_v_2_(t) = k_12_ v_1_(t) − k_21_ v_2_(t) (4)

with initial conditions v_1_(0) = c^(f)^ and v_2_(0) = 0. Where k_12_ and k_21_ are first-order rate constants, k_10_ is the elimination rate, v_1_(t) is the molecular concentration in the blood vessel network, and v_2_(t) is the concentration of information molecules when they reach nanonetwork. k_10_ is the elimination rate and v_el_(t) is the function of k_10_. The elimination rate represents the number of molecules that undergo biochemical modification, phagocytosis, elimination by liver or adhesion, and absorption by non-targeted sites, during the propagation process. Generally, the rate constants depend on the concentration difference between the blood vessel network and nanonetwork, the size of the aperture through the endothelial cell network, and the properties of the diffusing information molecules [42].

### 3.2. Bio-Electro Transduction Unit

**Figure 4:**
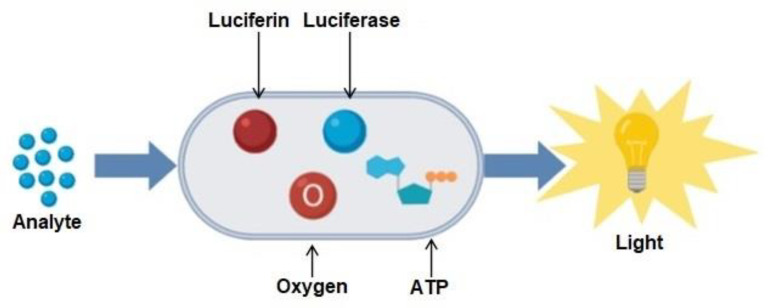
Representation of the bioreporter as a cellular structure, in which information molecules diffuse and a reporter protein (LU) is produced and undergoes a bioluminescence reaction in the bioreporter.

This unit presents reverse communication, i.e., detection of biochemical signals in the blood vessel network and converting it to an equivalent electromagnetic signal. A visual illustration of bioreporter activity is presented in Figure 4. 

This unit works on the principle of bioluminescence reaction. The conceptual model of this unit is a ligand–receptor transdermal system whose nanopores are extended into the blood vessels. The ligands here can be thought of as information molecules that circulate the blood vessel network and are detected by complementary receptors/nanopores. This ligand–receptor pairing results in the production of an equivalent electrical signal. Typically, luminescent materials are used to convert chemical signals into optical signals so that they can be detected by external devices. Bioluminescence is a phenomenon where a chemical reaction produces an electronically excited substance, called an enzyme-substrate system. The enzyme is Luciferase (LU), and the substrate is Luciferin(L) (from Lucifer in Latin “Light-bringer”) [43]. Bioluminescent molecules such as LU catalyze a chemical reaction with L in the presence of oxygen, which consumes chemical energy e.g., Adenosine triphosphate (ATP). ATP is a central metabolite that plays the role of an energy transfer molecule, a phosphate donor, as well as a signaling molecule inside the cells and produces light. The resultant light in turn is detected by a highly sensitive light sensor and is transduced into an electrical signal afterward. Bioluminescence intensity factor *I* can be expressed in the form of the Michaelis-Menten mechanism as follows [5]:

l(t) = a_l_.l_a_.atp/(atp + a_M_) (5)

where a_l_ is the catalytic reaction, l_a_ is the concentration of LU, atp is the concentration of ATP and a_M_ is the Michaelis-Menten constant. Let I_0_ be the bioluminescence intensity, hence when I(t) ≥ I_0_ the transmitter is in the ON state, and, if I(t) ≤ I_0_ the transmitter is in the OFF state. Bio-electro transduction unit receives signal from nanonetworks in the form of molecular concentration. The concentration of information molecules may change during the propagation time i.e., from nanonetwork/designated tissue into the blood vessel network and reaching the bio–cyber interface. This rate of change of molecular communication of the bio-electro transduction unit can be expressed through the equations below [5]:

c_(r)_ (t) = k_l_ w_1_ (t) (6)

d_t_w_1_(t) = k_21,r_ w_1_(t) − (k_12,r_ + k_10_+ k_l_) v_1_(t) (7)

d_t_w_2_(t) = k_12,r_ w_1_(t) − k_21,r_ w_2_(t) (8)

with the initial conditions w_1_(0) = 0 and w_2_(0) = m_0_, where m_0_ represents the molecular concentration released from nanonetworks. k_12,r_ and k_21,r_ is reverse equivalents of kinetic constants k_12_; and k_21_ respectively and k_l_ is the detachment constant of ligand–receptor pairing.

## 4. The Proposed Methodology

In the present section, we discuss the methodology employed for anomaly detection using deep learning technologies in bioluminescent interfaces of IoBNT systems. The proposed scheme comprises the following components: (i) *data generation and pre-processing* of bioluminescence parameter values, (ii) *neural network structures*, (iii) *experiment testbed,* and *measurement metrics*. The scheme monitors in vivo bio interface parameters to discriminate and identify anomalous operations. Using in-body parameter profiling allows higher accuracy in the classification of anomalies generated by external attack vectors compared to earlier studies solely relying on in vitro metrics such as bioFET electric voltage and current used [25], which may significantly vary due to electromagnetic interference and/or system faults, resulting in discrepancies. The following sub-sections detail each component of the proposed methodology.

### 4.1. Data Pre-Processor

The data pre-processor module accounts for collecting and sanitizing data. The parameters employed in the proposed scheme are unique to bio-luminescence-based cyber interface [4]. This work uses a synthetic dataset that consists of 12 key parameters based on existing literature in bioluminescence-based bio–cyber interface proposal for IoBNT [5,20,24]. The usage of synthetic data is a necessity due to the novelty of molecular communication using bio–cyber interfacing in IoBNT devices, which is yet to be fully realized practically. The data for this work depends upon the typical output generated by a bioluminescence-based bio–cyber interface. As described in Section 3, bio–cyber interface consists of two units: an electro-bio transduction unit and a bio-electro transduction unit. Each unit generates an output which is expressed in Equations (2) and (5). The performance of the IoBNT system depends on the output of bio–cyber interface units. Various parameters are required to derive these output equations. The description and value ranges of these parameters are presented in Table 2. These parameters are unique to bioluminescence-based bio–cyber interfaces and inappropriate values of these parameters represent anomalies in the system. The target is to ensure that appropriate values of these parameters are employed in the IoBNT system operation in order to achieve the required output. It is desired to find a set of parameters with minimal elements (features) whose values can be adjusted to ensure efficient communication. These parameters will be used as features to train our neural networking models. In the simulation, values of evaluation parameters used are taken from the literature and scaled up or down in the same order. A brief description of parameters and possible consequences of their abnormal values (out of the given range) are provided in Table 1 and described below:

Ψ_0_ is the concentration of message molecules encapsulated in liposomes, injected by an electro-bio transduction unit. A sufficient value of 0 ensures that accurate information is delivered to the targeted site. Tampered or manipulated values of this parameter, in (2), result in malfunctioning of nanomachines at the target site.

k_10_ is the elimination rate that elucidates the loss of information molecules due to the adhesion process, reaction process, absorption by non-targeted sites, phagocytosis, and elimination by the liver. Increased values of this parameter, used in (3), indicate that system might be under blackhole attack and information molecules are attracted by some malicious nanosystems [22,23].

a_M_ is the Michaelis-Menten constant. This parameter is an essential cofactor for ATP and its intracellular availability is dynamically regulated. It is observed as a weak parameter here as there is little effect of this constant on the bioluminescence intensity compared to other parameters as given in (5).

a*_l_* is the catalytic reaction constant. This parameter aids in adjusting the bioluminescent intensity. 

ATP is the concentration of adenosine triphosphate (ATP) and it is an important parameter as its appropriate value contributes to the bioluminescence intensity I0, which turns on the transmitter.

m_0_ is the molecular concentration parameter. It should be high enough to turn ON the transmitter and low enough to guarantee that the drug concentration does not reach toxic levels around healthy cells.

λ is the release rate constant and an important parameter. It is necessary to realize complete control of the drug release, from the initial release to a suitable release rate to ensure the best delivery profile for maximizing the treatment benefits while minimizing the amount of drugs in the other parts of the body.

k*_l_* is the ligand–receptor binding constant. k*_l_* is a crucial parameter as it represents whether the ligand–receptor bond happened or not. Inappropriate values of this parameter in (6), might indicate congestion, due to hits of ligand molecules with already busy receptors.

k_21,r_ is the forward rate constant. k_21,r,_ and other rate constants i.e., k_12_, k_12,r_ and k_21_ depends upon the concentration difference in the blood vessel network and nanonetwork, size of endothelial cell separating the blood vessel network and nanonetwork, and properties of the diffusing molecules. A sufficient value of this parameters indicates that more information molecules are received by the bio–cyber interface, resulting in higher bioluminescence.

Data set quality influences deep learning classification results. In the present work, data from simulated in vivo parameters of bioluminescent interfacing summarized in Table 1, will be used using binary classification. Binary classification will require anomalous or normal labeling of generated data during experimentation. Data will be split for testing and training, with a lower proportion of anomalous (attack) data conforming to realistic scenarios and to provide an accurate estimation of classification ability.

### 4.2. Neural Network Structures

The present work utilizes deep learning methods to analyze anomaly detection capability using bioluminescence intensity parameters of BBI. We first employ the convolutional neural network (CNN) algorithm offering lower latency during training and then offering weightage adjustment after setting initial parameters. The CNN allows the reduction of higher dimensionality using maximum pooling on features and indicates the most suitable feature traits. After testing CNN, recurrent neural network (RNN) structures are used to extract temporal characteristics of data. During extensive validation testing is done to observe the efficiency of different models and to devise a hybrid structure providing maximum classification benefits. The network structures and respective construction are discussed in the following sub-section.

Convolutional model: CNNs offer optimality in feature extraction at relatively lower latency through the creation of associations within data. Traditional implementations use the entry-level input layers to determine as well as learn features while secondary layers can derive more complex characteristics, representing data correlations. Convolution layer kernels filter features, each iteration producing a graph of features provided by each kernel. A pooling layer can be used for feature compression and selection, and the network converges on a final layer allowing connectivity to combine and categorize the model.

The complexity of CNN layers increases with the number of input parameters (exponentially) and can reduce network performance. The error rate is calculated using loss functions. Convolution operation depends on the number of kernels that produce proportional output, the step size determining the amount of data processed in any given internal and the filling factor to smooth out the learning. The convolution operation is given by (9).

O_(I,j)_ = Σ_(x=0)_ Σ_(y=0)_ K_(x,y)_.A_(q–−x,j–−y)_ (9)

**Figure 5:**
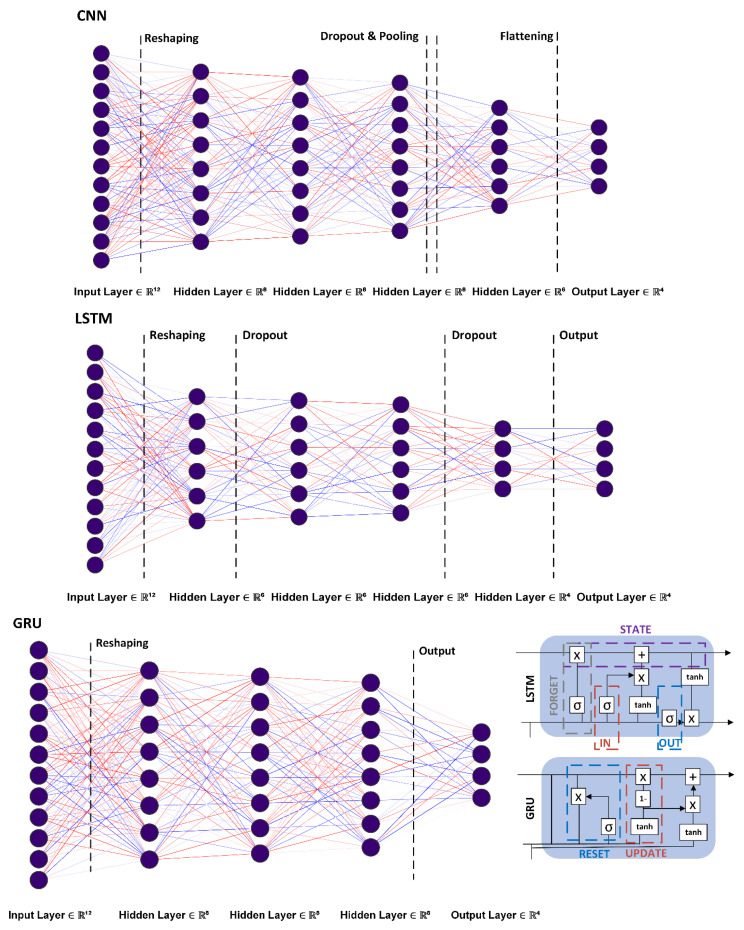
Neural network structures: CNN, LSTM, GRU, and RNN internal cell structure.

In total, the present study used six convolution layers, having a kernel size of 4 × 4 providing feature extraction capability. The first CNN layer has a relatively lower number of only twelve neurons for initial data processing. Successive layers provide 64 neurons each to improve classification. A dropout layer is added afterward to deal with overfitting, a common challenge in CNN. The maximum pooling layer provides feature differentiation, while the convolution layer processes the final set of features, compressed, and mapped by the dense layer at the end. A visual description of the employed CNN structure is provided in Figure 5. 

***Recurrent model:*** RNN models incorporate interconnected neurons in each layer so that the output of preceding layers is taken into consideration while computing the input to subsequent layers. Stateful structure and feedback mechanism allow RNN to understand temporal characteristics for anomaly detection [39]. RNN however, suffers from the vanishing gradient problem, and therefore, practical applicability on larger data series may require the use of long-short term memory (LSTM) and gated recurrent unit (GRU) implementations. In the present study, we utilize LSTM and GRU models using similar network structures and operational values. Data is input to the first layer, succeeded by the dropout layer adding an element of uncertainty for better training. Models and the individual cell structures are illustrated in Figure 5. Fewer neurons in hidden layers are used in LSTM (36) compared to GRU (64) in the hidden layers, as the former requires a greater number of training parameters and results in higher training latency. The dense layer is the final processing primitive in either model. The *sigmoid* activation is used in GRU and LSTM for filtering and output is sent using a tangential function (*TanH)* [38]. The LSTM and GRU operations are represented by (10)–(13) and (14)–(16) respectively. 

*Long short-term memory (LSTM) model input and variables.*

i_t_ = σ(W_i_[h_t−1_, x_t_] + b_i_) (10)

o_t_ = σ(W_o_[h_t−1_, x_t_] + b_c_) (11)

f_t_ = σ(W_f_[h_t−1_, x_t_] + b_f_) (12)

c_t_ = tanh(W_C_·[h_t−1_, x_t_] + b_C_) (13)

*Gated recurrent unit (GRU) model input and variables.*

r_t_ = sigmoid(W_r_[h_t−1_, x_t_] + b_r_) (14)

z_t_ = sigmoid(W_r_[h_t−1_, x_t_] + b_z_) (15)

r_t_ = sigmoid(W_r_[r_t_.h_t−1_, x_t_] + b_c_) (16)

The sigmoid, rectified linear unit (ReLU), and general loss functions are given by (17)–(19), respectively.

g(z) = 1/[1 + e^−z^], g’(z) = g(z)(1 − g(z)) (17)

g(z) = max (0, z), g(z) = {1, z > 0, 0 otherwise(18)

L(W) = 1/n ∑_k=0→n_ y^(i)^log (f(x^(i)^; W)) + (1 − y^(i)^) log (1 − f(x^(i)^; W)) (19)

In (18), (19), y^(i)^ is the actual value while f(x^(i)^; W) is the prediction.

### 4.3. Experiment and Measurements

The present section discusses the experiment specifics of the study, detailing data generation, the tools used for implementing deep learning algorithms, and the measurements recorded.

#### 4.3.1. Data Processing

**Figure 6:**
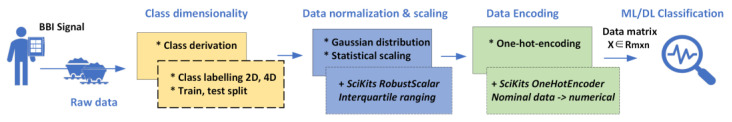
Data processing—primary steps.

The primary steps involved in *data processing* involve *class derivation and labeling*, *value normalization,* and *encoding* for improved model fitting. An illustration of the main steps is illustrated in Figure 6, and these are discussed in detail as follows. 

***Step 1: Class-derivation and labelling:*** In total 7,985,539 records each having twelve feature values were generated using the bioluminescence parameters given by (1)–(8), and equally split for training and testing summarized earlier in Table 2. As per existing literature, out-of-bound variation in a minimum of three out of twelve (or greater) parameters of any given input vector constitutes an anomaly [2,7,20,33,41]. Therefore, in two-dimensional (2D) class distribution abnormal parameters constituting more than ~25% of an input vector result in final class derivation being *anomalous*, and otherwise *normal*. Using 2D binary distribution, 6,866,701 samples represent *normal* and 1,117,829 *anomalous* vectors, indicating an overall ~86:14 probability split between normal and anomalous data as given in Table 3. However, having uniform 2D class labeling does not completely account for the scarcity of instances where normal in vivo channel communication presents realistic outliers, and the resulting operation is not necessarily anomalous due to realistic variations in molecular traffic [2,7,15,23]. In 4D classification, the normal-anomalous (NA) class realizes the normal operation even though the threshold parameter varies outside the threshold; and the anomalous-normal (AN) class represents abnormal operation despite parameters ranging within the stipulated threshold. NA and AN vector instances constitute ~3.4–3.9% of normal and anomalous vectors based on existing studies [7,8,21,22]. The resulting data is stored in matrix X ∈ R_mxn_ where *m* represents the number of samples and *n* is the number of variables (twelve). Vector L ∈ R_mx1_ provides a class label (string). 

**Figure 7:**
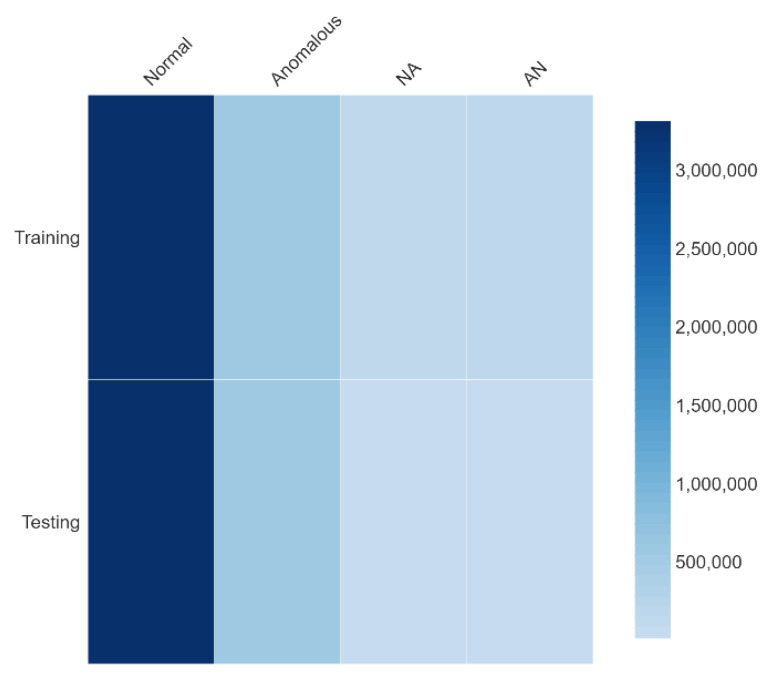
Density distribution—bioluminescence data.

***Step 2: Data normalization and scaling:*** The parameter density distribution of generated data (training and testing) for each of the 4Dclass-labels is provided in Figure 7, and an online dataset representation is provided in [44]. The data follows a Gaussian probability distribution given by (20) and is symmetric about the mean.

f(x) = 1/σ √(2π) × e^−1/2(x−μ/σ)^2^ (20)

In (20), x represents the class variable being examined, f(x) is the probability function, μ is the mean, and the σ standard deviation. The generated data follows a Gaussian distribution and does not require smoothing filtration [37]. However, normal BBI operations may encompass signal fluctuations, and nonlinear responses resulting in outliers that can affect classification accuracy. Although these outlier values on data distribution edges (NA, AN) have a low occurrence probability, yet capable of over-representation during training. To avoid model skewing inherent relative variability in BBI parameters requires redressal using statistical methods to achieve high throughput during classification. Complementary techniques such as local-neighbor density deviation (LOCAL), isolation using modeling (ISO), and elliptic envelope (ELLI) can cause decision edge confusion, data scattering, and computationally intensive [27,28,37]. Statistical outlier detection using robust scaling is used in the present study to maintain data characteristics while reducing data scattering with minimal overhead. Using the median (50th percentile), and 25th and 75th percentiles, in robust scaling each parameter median is subtracted and divided by the interquartile range (IQR) [32]. This ensures resulting variables conform to zero mean, median, and standard deviation of one while remaining non-skewed by outliers and maintaining relative relationship with other variables (parameters). Outliers depicting normal class with MC channel variation are, therefore, upheld and contribute to the discriminatory ability of the resulting classifier in recognizing false positives, as well as negatives. Robust scaling for the present experiment is achieved using Python SciKits RobustScalar class, setting with_centring argument centered to zero, and with_scaling set to IQR, and both values defaulting to True, as listed in Table 4 [37]. Given the stochastic ability of algorithms differences in numerical precision are possible in iterative runs during classification and as such the percentile ranges for scaling were varied (20–80, and 30–70) to derive optimal accuracy. Compared to similar studies, the proposed data normalization technique fits well on low-dimensional tall data offering minimum scattering while exploring full (traffic) characteristics for optimal classification [28,29].

***Step 3: Data encoding:*** The data composition matrix (X ∈ R_mxn_) comprises twelve continuous data parameters (n = 12) with range-bound probable values, as well as one nominal variable (L) representing 4D class labels without any intrinsic ordering. One hot encoding using SciKits OneHotEncoder class is a used for numerical conversion of this nominal data before the application of classification algorithms [37]. Since the class labeling does not have any intrinsic order, hence ascending encoding (0–3) cannot be used otherwise ML algorithms will give priority to higher numbers. Each category is hence, transformed into a new column and given a binary range (0,1) depending on the occurrence of the respective label. Post-transformation dataset, therefore, results in a greater number of features due to one hot encoding of traffic class labeling. Compared to other nominal conversion schemes such as Bag-of-Word (BoW), and MinMaxScaling, one hot encoding provides a single-stage conversion suitable for BBI parameters relying on substantially lower computing capability favored in IoT environments [29,31]. The pre-processed dataset is subsequently input to neural algorithms using control variables described in the next sub-section.

#### 4.3.2. Neural Network Structures and Control Variables

The neural network structures tested include CNN, LSTM, and GRU. The model structures used are provided in Figure 5. The CNN model comprised a single input layer containing 12 neurons, four hidden convolutional layers each containing 64 neurons, and a maximum pooling, dropout, and flattening layer. LSTM also comprises one input layer (16 neurons) as well as a dropout in hidden layers. The GRU model has an input layer (12 neurons), and three GRU layers having a greater number of neurons (64, 128, 156) and dropout in hidden layers. As discussed earlier, GRU has an overall greater number of neurons in comparison with LSTM models, but the operational values used are smaller. The dropout for all models was set to the commonly used value of 0.5. For activation, ReLU was employed as preferred in earlier studies [34,37]. The output layer uses federate AI (FATE) [45] and is classified using softmax activation [37]. Control variables used for experimentation are listed in Table 4. Training and testing on each neural network model are conducted using an epoch of 10 on a trial-and-error basis with the same input–output dimensions and class (category) labeling. To reduce overall cost-function Nadam-Nesterov-acceleration adaptive moment estimation (gradient descent) is employed using Keras [46]. Nadam optimizes model learning and classification by summative exponential reduction of floating averages on preceding and successive gradient values. For computing the overall loss function, seeking to minimize the training phase, categorical cross entropy available in Keras is used [47]. The experiment was carried out on a researcher’s machine with an Intel Core i7-960 16 MB chipset at 3.2 GHz with RAM 64 GB. The operating system used was Ubuntu 18.04.5 (64-bit) hosting a testing platform using Keras 2.4.0 and TensorFlow library 2.8.0. [48]. The designed neural network models were evaluated on the generated dataset to record accuracy, precision, recall, as well as the false positive and negative rates given in Table 5.

## 5. Results and Discussion

The present section discusses the evaluation results of proposed deep learning structures from a quantitative and qualitative perspective as well as an analysis of the optimum classification model.

### 5.1. Quantitative Results

The recorded efficiency of each model for four-dimensional parameter classification using the network structures and system variables discussed earlier is given in Table 6. The quantitative results are systematically analyzed to determine the best classifier from an operational perspective considering training latency, testing duration, as well as reported accuracy and precision. CNN is considered first, with additional structures subsequently added to the testbed for evaluation. For comparison purposes, a 1D shallow CNN is also considered.

The time complexity of each neural network structure is considered to indicate the time involved in training and testing the classifier. Each classification model was considered under ten successive training iterations. The shallow network outperforms all others in terms of latency which is expected. Each training iteration of the shallow neural network (1D-CNN) was recorded at approximately 2.7 s having a total training time of 26.81 s. Deep-layered CNN training time was the lowest among all remaining non-hybrid and hybrid classifiers at 215.89 s. CNN processed 399,226 samples for each training iteration in ~21.58 s. The LSTM consumed 78.56 s and GRU 92.12 s for each training iteration recording a total training time of 785 s and 921 s respectively. Among the hybrid models, the CNN + LSTM model offered the advantage of faster extraction of relevant features (due to CNN) and a higher cross-dimensionality analysis (provided by LSTM) at a relatively lower training time of 315.6 s compared to the CNN + GRU model at 468.12 s. During testing and validation, the 3,992,271 samples were completed in ~117 s by CNN, ~257 s by LSTM, and ~312.89 s by GRU. Similarly, the CNN + LSTM consumed around 129.31 s during testing compared to the CNN + GRU, which recorded 221 s.

**Figure 8:**
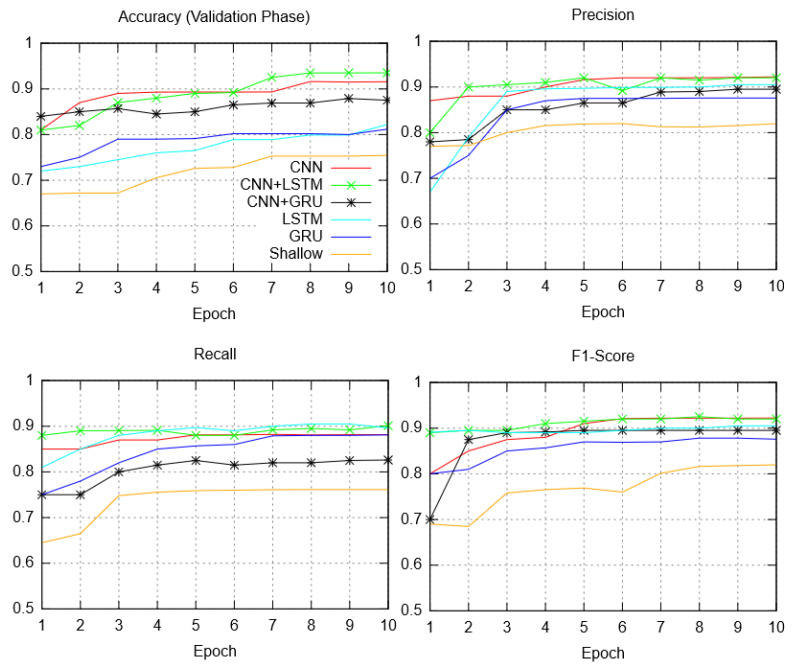
Quantitative results: accuracy, precision, recall, and f1-score.

Accuracy, precision, recall, and f1-score for each of the neural network models is demonstrated in Figure 8. Among non-hybrid classifiers, the CNN reported highest accuracy (91.56%) and precision (92.21%) when compared to LSTM and GRU classifiers. Similarly, the false positive rate (1.45%), recall value (88.15%), and f1-score (0.92) were optimal among non-hybrid models. Among all classifiers the CNN + LSTM network reported the highest accuracy (93.51%), while the precision (91.81%) was slightly lower in comparison with CNN, the recall (90.11%) and FPR (1.10%) was again optimal while the f1-score was only slightly lower than CNN. The CNN + GRU structure fared well in accuracy (87.52%), and precision (85.61%) with 1D CNN (shallow), LSTM, and GRU classifier.

### 5.2. Qualitative Observations

Deep neural networks aid in data dimensionality reduction by learning features of interest applicable to real IoBNT operation. From the empirical results, it is witnessed that the overall training and testing time of the CNN model is better in comparison with the other non-hybrid and hybrid classifiers. The overall efficiency of CNN is also significantly high albeit lower than the CNN + LSTM hybrid. As discussed earlier, LSTM and GRU models are from the recurrent neural network (RNN) family having different operational focus compared to typical CNN structures. RNN-based models help in extracting interdimensional correlations in data to identify the anomalies in bioluminescence operational parameters. It was observed that the GRU and LSTM training and validation require a longer time, however, the efficiency in comparison was relatively lower. The relatively low accuracy of RNN models in the evaluation is attributed to over-fitting and the absence of high visibility time-related parameters in data. LSTM and GRU models, therefore, did not produce the highest classification accuracy in standalone non-hybrid mode. It was also observed that the convergence time of LSTM and GRU was quite similar, with the classification accuracy and precision of LSTM being better. The shallow model reported minimum time latency, but the accuracy and precision were the lowest among all models. The shallow model appeared to provide significant data fitting, but the absence of a deep(er) neural network affected the overall generalization in discriminating anomalous traffic parameters. The hybrid models performed well in comparison with LSTM, GRU, and the shallow model. The CNN + LSTM hybrid provided the highest accuracy, with precision fractionally less than CNN. The remaining metric values of the CNN + LSTM outperformed all classification structures. 

### 5.3. Hybrid Structure: Analysis and Discussion

**Figure 9:**
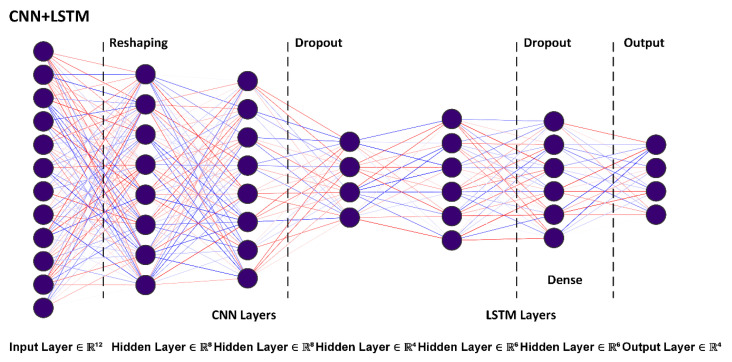
Hybrid neural network model.

The hybrid CNN + LSTM proved effective with respect to classification results obtained for non-hybrid, shallow, and hybrid neural networks. The CNN part offers low-latency feature extraction capability by using iterative convolutions on traffic parameters, allowing a relatively accurate weight representation using small-sized kernels. Convolutional layers are succeeded by the LSTM structure. The complete model is depicted in Figure 9. Architectural insight into each layer of the hybrid model is detailed below.

Convolutional (entry-level) input layer: input layers comprise twelve neurons for data processing and early feature selection. Employing a relatively lower number of input neurons aligns with a fundamental IDS/IPS requirement to allow faster initial processing with complex data correlations being extracted in secondary (hidden) layers [23,37].

Hidden (secondary) layers: the hybrid model used two (fully connected) hidden layers having sixty-four neurons each, and a 4 × 4 kernel size to allow comprehensive feature extraction. Using two hidden layers, with a relatively higher number of neurons compared to the input and final output layers results in feature filtering for large dimensional BBI input data [34,39]. ReLU (non-linear piecewise) activation is used to reduce the vanishing gradient issue and prevent an exponential increase in computation to operate the DL network and improve deployment feasibility [34,37]. ReLU function, hence, helps the hybrid model to learn faster and optimizes performance. 

Pooling layer (deletion): pooling layers which provided the means to reduce feature dimensionality in independent CNN earlier, are not used in the hybrid model. This purposely allows the filtering of low-intensity temporal BBI features and provides greater invariance before further processing in LSTM layers [49].

Dropout layers: dropout layers are used in fully connected layers, to address scholastic regularization and to limit model overfitting. Dropout layers are included at the end of CNN layers, and after LSTM input add an element of uncertainty for improved model training. The present implementation used a typical dropout value of 0.5, to regularize model over-fitting and minimize weight learning in each iteration [14,16].

Dense layer (LSTM): Dense layer uses sigmoid activation given in earlier in (13), changing the output to follow a tangential function [38]. Sigmoid function guarantees any neural unit output (weighted sum of inputs) to conform to 0 and 1, before being input to (any) subsequent layer. ReLU activation at each preceding layer ensures that sigmoid is only applied at the end in the hybrid model to avoid saturation at data extremes during training which would reduce model learning ability. 

Output layer: the output layer provides final feature differentiation, compression, and mapping output to one hot encoding (class label) at the end. Softmax function is used in the output as a last activation to predict multinomial probability distribution [36]. The function assigns a decimal probability to each of the four BBI traffic classes (adding up to 1.0), essentially reducing the overall training convergence latency of the hybrid model.

Optimization function: Nadam is used to minimize exponential floating averages on all preceding and subsequent gradient values using Keras [46]. The optimization ensures better overall loss function and faster convergence reducing the training phase. This is an enhancement to the Adam optimizer used in several earlier classification studies and allows better anomaly detection capability in multi-dimensional parametric data such as IoBNT BBI.

The final structure, is an ensemble of convolutional and recurrent long-short-term layers, allowing single-stage processing. We consider the performance of this hybrid model in the context of individual CNN, and related studies as well as highlight the inherent operational challenges in the following sub-sections.

#### 5.3.1. Performance Comparison

**Figure 10:**
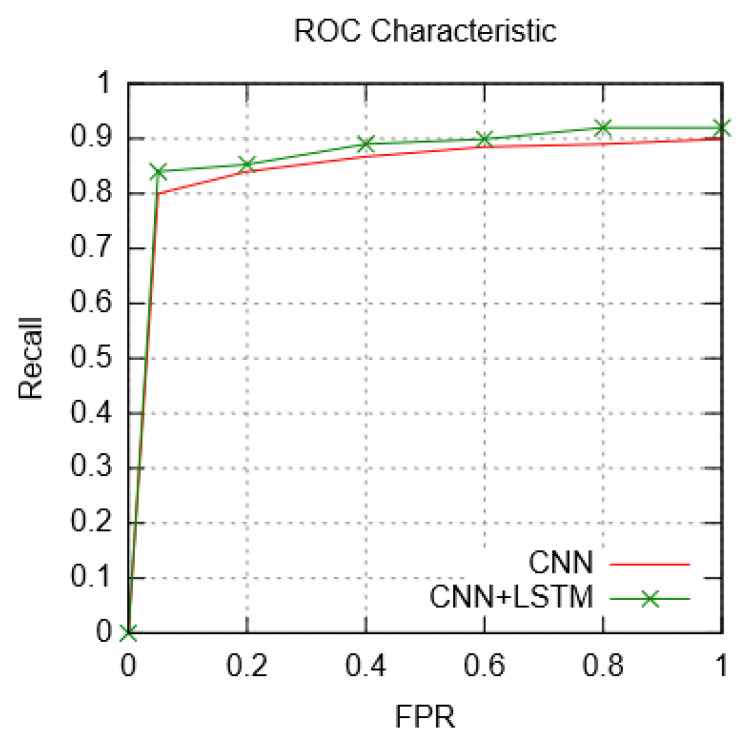
ROC Curve: CNN; CNN + LSTM Hybrid model.

To compute the overall loss function, signifying a reduction in training phase, categorical cross entropy from Keras was used [47]. The receiver operating characteristic (ROC) curve for the optimal classifier in comparison with CNN is given in Figure 10. The resulting differences between the two models are subtle at certain thresholds. Although the overall difference in recorded metrics for the CNN, and CNN + LSTM model was minimal, the LSTM layer in the latter provides the additional benefit of extracting temporal correlations present in the in vivo traffic diffusion parameters such as the cumulative concentration rate released molecules (Ψ_0_), the diffusion rate of molecules (m_0_), and the drug delivery release rate (λ). Compared to ML, and DL models for generic and IoBNT-interface-specific anomaly detection classifiers in [23,24,25,26,27,28,29,30,33], the optimal hybrid model can classify anomalous parameter traffic by dynamically reducing feature dimensionality which would be manually intensive if using traditional ML. Summarization of key differences and advantages of the proposed hybrid model over complimentary schemes are detailed in Table 7. Zafar et al. [23] provided an authentication method for IoBNT interfaces employing channel impulse response (CIR) profiling to isolate anomalous control signals. El-fatyany et al. [24] relied on binary-phase-shift-keying (BPSK) modulation to encryption system-IoBNT communication keys. These early non-ML reliant schemes allowed limited toleration for noisy signals affecting in-vivo TDD, while also being computationally intensive due to inherent multi-stage encryptions. Among ML/DL classification use-cases in IoT, and IoBNT domain, the primary constraint has been manual or multi-stage feature engineering as is the case in [25,26,27,28,29,30,31], as well as limited dataset diversity as in [33]. In the IoT domain, utilize semi-hybrid classification strategies to detect network anomalies. Classifiers proposed by Abusitta et al. [26], Rosero-M et al. [27], Chander et al. [28], and Hameed et al. [29], rely on either extensive unsupervised ML-based feature engineering followed by neural network training (semi-hybrid models), use multi-stage process(es), or allow only binary traffic classification, limiting practical realization of these classifiers in IoBNT-specific interfacing for timely anomaly detection. The present hybrid (CNN + LSTM) model, in contrast, allows for automated feature processing and selection using a neural network structure. Extensive multi-stage feature engineering prior to DL application results in significant computational overhead which would be counter-intuitive for deployment in IDS, and IPS if re-training were required. Furthermore, supervised feature selection represented in prior schemes necessitates a new cycle of data collection, annotation, and training for updating classifiers [26,27,28,29,30,31]. However, such a training primitive is not applicable for the proposed hybrid since the addition of new data would only require preparation (pre-processing), with convolutional layers performing necessary feature selection. The operational considerations and performance challenges of the proposed model are considered in the following sub-section. 

#### 5.3.2. Operational Considerations and Challenges

The primary limitation of the CNN + LSTM hybrid model is visible in the training and testing latency (~120–316 s) which is marginally higher than the non-hybrid CNN classifier (~117–216 s) as depicted in Table 5. The present evaluations on the test-data set reflect the comparative accuracy and false-positive ratio of a hybrid classifier to be 93.51%, and 1.10% over CNN which is 91.56% and 1.45% respectively. Real-time deployment of the CNN classifier in an IoBNT-specific intrusion detection and prevention system (IDS, IPS) would, therefore, benefit from low alarm trigger latency, albeit decrease in accuracy. Lowered accuracy may trigger a higher number of false positive alarms, enabling mitigative measures which in turn would affect the molecular channel communication and subsequently disrupt intended drug delivery. Correspondingly, using CNN along with LSTM realizes greater accuracy (by ~2%), with a partial increase in anomaly detection time (around ~11%) as observed for the present dataset. Factors impacting the effectiveness of CNN + LSTM, CNN, or any other monolithic and hybrid DL model can, hence, mainly be analyzed across two different operational strands (i) alarm latency, and (ii) reported accuracy. These factors are in turn influenced by the availability of realistic, diverse, and voluminous datasets pertaining BBI-IoBNT scenario. Time-rich features and inherent correlation among different parameters in network traffic typically require memory-retaining structures (such as LSTM), which when combined with convolutional models allow for better overall accuracy. Realistic IDS/IPDS deployments, therefore, need to realize the peculiarities of specific traffic datasets, implementation (edge, fog, cloud) with respect to time constraints, and adequate testing to determine the optimal configuration of the hybrid structure. From the perspective of operational safety reduction in false positives is significant in in-vivo drug delivery in (any) IoBNT healthcare primitive and may consequently be traded off for a partial increase in event detection time complexity [1,12,13]. Reducing FPR, allows for greater streamlining of delivery and monitoring of MC-based drugs, without falsely triggering and enabling mitigation measures, and degrading performance that may negatively impact the intended outcome of healthcare operation(s). Novel system engineering deployment primitives for the proposed hybrid model are discussed in the following section.

### 5.4. System Engineering: Deployment Primitives

The CNN model and the CNN + LSTM hybrid have demonstrated high accuracy, with the hybrid faring better in terms of overall performance. It is important to consider the practical applicability and integration of the proposed classifiers for the bioluminescent bio interface of IoBNT in intrusion detection systems (IDS). The hybrid model devised in the present work utilized hardware and software resources described earlier in Table 4, implemented on a researcher (computing) machine having the capability to process data and deploy classifiers; a setup challenging to implement in patient homes, or medical facilities which do not have adequate computing or personnel. IoBNT paradigm and bioluminescent interfaces recommend smaller real-estate, as well as minimum computing power for human (and patient) friendly integration to offer real-time anomaly detection capability arising due to cyber-attacks coupled with the longevity of practical operation. We explore the possibility of using conventional and emerging computing paradigms to address the practical deployment of a hybrid DL-based IoBNT anomaly classification proposal as follows.

Fog and cloud computing: A possible solution to timely processing and deployment of intrusion detection monitors is the use of fog-based classifiers, coupled with back-end cloud computing for data processing and updating of DL structure to address unseen and new(er) threats [20,34]. The anticipated challenge would be addressing the feasibility of a fog computing server/node in patient premises, with satisfactory Internet connectivity to back-end monitors. Such a scheme primarily relies on software-based systems for data processing, monitoring, and intrusion (anomaly) detection. 

Neuromorphic computation: A complimentary system deployment approach is to employ hardware-based processing for real-time anomaly detection through in-situ deployment of a derived hybrid DL classifier in a bioluminescent bio-cyber interface using a neuromorphic (chipset) module [50]. Emerging neuromorphic computing chipsets such as Loihi by Intel [51] and Systems of Neuromorphic Adaptive Plastic Scalable Electronics (SyNAPSE) TrueNorth program by IBM [52,53] comprise programmable neural network learning engines that can self-train as well as adapt to external model parameters. Loihi uses spiking neural network (SNN), and neural spike through directed synapses. The learning engine can be tuned by updating synaptic weights and spike timings using custom learning rules based on required network parameters through a 4-bit microcode and an API. The chip, therefore, allows programming according to externally derived classifiers such as the hybrid DL network structure proposed in the present work. Additionally, learning can also be done on-chip using flexible supervised, unsupervised, reinforced, and configurable neural networking architectures using a Python-based API, similar to Python Neural Network Ensemble (PyNN) [54]. SyNAPSE by IBM is based on a multicore neural network chip. The chip comprises 4096 cores and over one million neurons, each neuron having 256 programmable synapses, conveying inter-neuron signals representing over 268 million synapses. Loihi and SYNAPSE architectures handle memory, computation, and communication in neuro-synapses. Hence, these chipsets are not bottlenecked by the power requirements of typical von Neumann architecture, proposing low-power, efficient intrusion detection classifier incorporation in IoBNT devices. Intel claims 1000 times the energy efficiency of Loihi over traditional computing power needed to train the same neural network. IBM claims the SyNAPSE (TrueNorth) ecosystem to have 70 mW consumption with a power profile of 0.01% to conventional processors. Low spatial requirements of Loihi2 (60 mm^2^) and SyNAPSE (~35 mm^2^) may offer integration into the IoBNT system eliminating the need for an additional local computing/fog node in the patient enclosure. On-chip learning capability results in more localized DL classifier updating reducing reliance on peripheral and cloud computing devices [55]. In theory, machine learning and monitoring via the same silicon, independent of (any) external cloud connections provide a very useful deployment paradigm in IDS.

**Figure 11:**
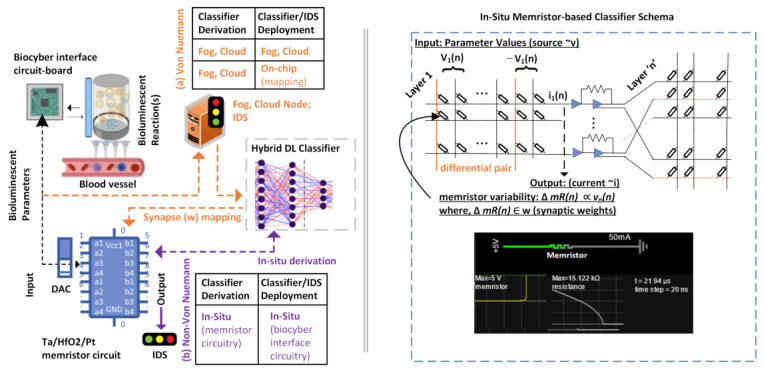
Neuromorphic hybrid DL classifier deployment using 1T1R memristor crossbars.

Analog memristor crossbars: Studies in neuromorphic computing have realized the implementation of neural network structures on cross-connecting circuits, where resistive circuits can be programmed to represent neural network structures at a fraction of the cost associated with commercial chipsets. Analog circuitry has been researched to successfully build on-chip neural network classifiers [56,57]. Multilayer neural networks have been shown for successful deployment on One Transistor One Resistor (1T1R) memristor-based crossbars [58]. We present a representative approach of employing analog (crossbar) circuits to implement the proposed hybrid DL-based intrusion detection classifier at the system level. Using Ta/HfO_2_/Pt memristors, the hybrid DL-based classifiers, such as one derived for attack classification and anomaly detection in the present study can be ported to hardware circuitry. A typical hardware implementation of the hybrid DL intrusion detection classifier influenced by earlier research is illustrated in Figure 10. Each memristor can be connected in series to a transistor forming a 1T1R structure, where multiple 1T1R arrays can be assembled to represent the multi-layer neural network structure illustrated in Figure 11. The number of input voltage sources directly corresponds to the number of parameter inputs (12 bio-luminesces metrics) to the CNN + LSTM classifier. The weight training (synapses) of the hardware circuitry would need mapping to the derived CNN + LSTM parameters at each neural layer. In the crossbar(s), each synapse (weight) is represented by the conductance difference between two memristors, and the weighted sum of input voltages (v_1_, ..., v_n_) is calculated at each cross-point. Integrated circuitry in the crossbar arrays reads current values at each cross-point, converting it to equivalent voltage to represent the activation function (which is non-linear). Ubiquitous availability of 1T1R, and on-demand foundry-fabrication of transistor arrays as depicted in Figure 11, are practically realizable. Furthermore, fully trained memristor networks can be integrated with analog-to-digital (ADC) and digital-to-analog (DAC) converters for peripheral integration with IoBNT devices. IoBNT modules such as the BBI bio-cyber interface can read analog signals, processed on fully trained memristor arrays according to the operational characteristics of the pre-built DL model, and further communicated to off-site monitors. Memristor crossbars embed the benefits of non-Von Neumann architecture while allowing lower power utilization at a fraction of traditional computing.

The derived hybrid DL anomaly detection classifier, therefore, can be used with different software and hardware primitives to fulfill intrusion detection requirements. While classifier derivation can be conducted on typical computing machines as demonstrated in the present work, IDS and real-time attack monitoring can benefit from hardware-based classifier ports. The suggested proposal of hardware porting of the built hybrid classifier, employing commercial neuromorphic chipsets as well as foundry-fabricated analog crossbar circuitry allows real-time inference, training, and anomaly detection in IoBNT (bio-cyber bioluminescence) interface security at highly economized power and real-estate costs. Using a chipset or circuitry of the scale of a few square millimeters allows a greater scope of inclusion in the bioluminescent interface module, increasing the ergonomic value of the design. 

## 6. Conclusions and Future Work

The present work proposed a hybrid deep-learning neural network structure to detect anomalous attack traffic and secure the bioluminescent bio-cyber interface (BBI) of the Internet of Bio-Nano Things (IoBNT) framework. During testing and validation, the deep learning-based classifier based on a CNN + LSTM hybrid structure reported an accuracy of approximately 93.51% in comparison with other models including standalone CNN, LSTM, GRU, and CNN + GRU hybrid (ranging between ~73–91.6%). It was observed that the CNN model marginally excelled in feature extraction latency (~117 s) relative to CNN + LSTM (~130 s). However, the later hybrid structure demonstrated higher discriminatory precision in classifying anomalous traffic by being able to account for temporal characteristics in traffic, provisioned by the LSTM part. The hybrid CNN + LSTM structure, therefore, allowed higher accuracy which is essential in real-time IDS deployments to minimize the impact of adversarial anomalous traffic through accurate identification and subsequent mitigation during in-vivo drug delivery and monitoring.

The present work also explored the utilization of neuromorphic chipsets, and analog circuitry to port the proposed classifier at the hardware level; aiming to economize power consumption, and spatial efficiency in ergonomic IoBNT healthcare deployments. In our future work, we will map the pre-determined classifier attributes (synaptic weights) on memristor crossbars as well as investigate the possibility of in-situ classifier training, to reduce resource consumption using non-Von Neumann architecture(s).

## Figures and Tables

**Table 1:** Related work and cross-connecting themes.

Research Domain	Scope of Work	Cross-Connecting Themes
IoBNT Systems [1,2,3,4,5,6,31]	Comprehensive survey, technology reviews, and use cases	Threat analytics, IoBNT interface, molecular communication, TDD
Attack analysis and mitigation [22,23]	Attack taxonomy (black-hole, sentry), countermeasures, authentication schemes	Threat analytics, IoBNT interface, OS fingerprinting, access control
BBI Security [24,25]	BBI authentication, design, decision tree-based IDS	ML, traffic classification, logistic mapping, TDD
BioFET Security [25,33]	C5.0, ANN, PSO-based IDS for BioFET interfaces	ML, Traffic classification, anomaly detection, deep learning
Redox Security [25]	Decision tree and regression-based IDS	ML, traffic classification, feature engineering
MC and in-vivo Security [20,21]	Security requirements, deployment	Threat analytics, access control, TDD
IoT (IDS, IPS) [26,27,28,29,30,32]	Neural network-based IDS for IoT environments (applications in IIoT, SmartCity, 5G networks)	ML, traffic classification, feature engineering, anomaly detection, deep learning, performance comparison, de-noising (data)

**Table 2:** Simulation Parameters.

Parameter	Description	Value
Ψ_0_	Cumulative concentration of released molecules	0.62–1.50 mL
k_10_	Elimination rate	0.172–0.472 min^−1^
a_M_	Michaelis-Menten constant	10–17 µM
k_12_	Kinetic constant	0.073–0.373 min^−1^
k_21_	Forward rate constant	0.00053–0.00103 min^−1^
a_l_	Catalytic reaction constant	4.1–4.4 × 10^−2^ µM
k_l_	Ligand–receptor binding constant	0.001–0.0015 min^−1^
Atp	Concentration of ATP	10–40 µL
m_0_	Concentration of information molecules	5–10 µM
Λ	Release rate	0.0104–0.0404 min^−1^
k_12,r_	Reverse Kinetic constant	0.00043–0.00103 min^−1^
k_21,r_	Forward rate constant	0.073–0.373 min^−1^

**Table 3:** Dataset Specification.

Distribution	Class Label	Training Data	Test Data
1 D	Normal + Anomalous	3,992,268	3,992,271
2 D	Normal	-	3,433,351
	-	Anomalous	558,917
4 D	Normal	-	3,316,617
	Normal {abnormal parameters > 25%}	-	116,734
	-	Anomalous	538,796
	-	Anomalous {abnormal parameters < 25%}	20,121
Total	-	-	6,866,701

**Table 4:** System Variables.

Property	Parameters	Comments
System	Core i7-960 16 MB 3.2 GHz 64 GB	Intel architecture DDR3L 1066
	Ubuntu 18.04.5	Kernel 4.15.0-135- 64
	Keras 2.4.0.	DL API (Python) [32]
	TensorFlow 2.8.0.	ML Library (Opensource) [33]
Data Processing	Data density distribution	Gaussian PDF
	RobustScalar (options)	SciPi toolkits using RobustScalar class [37]
	with_centering	controls value centering at 0 (median is subtracted) and set to default True.
	with_scaling	controls IQR scaling (standard deviation of 1) and set to default True.
	quantile_range	Tuple (0–100), default (25, and 75)
	Data encoding	Non-intrinsic traffic labeling using the OneHotEncoder class [37]
Neural Network Convolutional/Recurrent	Rectified Linear Unit (ReLU)	Non-linear (piece-wise linear) activation function used in DL classifiers [37]
	FATE/FederatedAI	The final layer in the DL model to aid logistic regression [45]
	Dropout = 0.5	Regularization method reducing over-fitting by minimizing weight learning per iteration [14,16,37]
	Softmax output function	The normalized exponential function used as the last activation in neural networks to normalize output [37]
	Optimizer = Nadam	Gradient descent algorithm employed to minimize cost function [37,38].
	Loss function = Categorical cross-entropy	Loss function to benchmark neural network reduction in the training phase [37,39]

**Table 5:** Metric Rubric.

Metric and Description	Calculation
Accuracy: The ratio is correctly identified and divided into total samples.	$Accuracy=\frac{TP+FP}{TP+FP+TN+FN}$
Precision: Positive predictions (correctly identified).	$Precision=\frac{TP}{TP+FP}$
Recall: Ratio of anomalies correctly classified to total samples.	$Recall=\frac{TP}{TP+FN}$
F1-Score: Metric defining mean value determined for P and R times two.	$F1score=\frac{2*P.R}{P+R}$
False Positive Rate (FPR): Ratio of anomalous to normal samples.	$FPR=\frac{FP}{FP+TN}$
Normalization factor: The data set is normalized using the max. and min. of input (column-wise), then to each data value.	$Z=\frac{z-min}{max-min}$
Time- Latency: The total time involved in training and testing a neural network	Training duration = TD Testing duration = TD’

**Table 6:** Result Evaluation—Quantitative.

Metric	Neural Network Structure					
	Non-Hybrid				Hybrid	
	1D CNN	CNN	LSTM	GRU	CNN + LSTM	CNN + GRU
TD (s)	26.81	215.89	785.61	921.24	315.6	468.12
TD’(s)	11.5	116.81	256.84	312.89	129.31	221.51
A (%)	73.42	91.56	82.24	81.17	93.51	87.52
P (%)	69.14	92.21	89.31	81.62	91.81	89.61
R (%)	76.15	88.15	89.62	88.11	90.11	82.61
FPR (%)	1.21	1.45	14.12	6.89	1.10	2.81
F1- (%)	81.98	92.21	90.51	87.58	92.01	89.51

**Table 7:** Anomaly classification models—model performance comparison.

Contribution	Domain	Classifier	Constraint(s)	Features
Zafar et al. [23]	IoBNT	Impulse response fingerprinting	Significant probability of incorrect detection in noisy data	CIR fingerprinting for anomalous control signal discrimination
El-Fatyany et al. [24]	IoBNT	Logistic mapping and BPSK modulation	Multi-stage encryption, in-vivo TDD disruption, compute overhead	BPSK session key encryption for IoBNT interface signaling
Bakhshi et al. [25]	IoBNT	ML (supervised learning—C5.0)	Manually crafted features, limited dataset, satisfactory efficiency	Security profiling BBI, BioFET, and Redox IoBNT interfaces
Abusitta et al. [26]	IoT	Semi-Hybrid: ML (un-supervised) + DNN	Unsupervised ML-based feature engg., 2D classification	De-noising traffic parameters followed by the NN application
Rosero et al. [27]	IoT	Semi-Hybrid: ML (unsupervised) + DNN	Multi-stage feature processing, computation overhead	kNN, NB, SVM, heuristics-based outlier detection, followed by RNN
Chander et al. [28]	IIoT	RNN (GRU)	Multi-stage metaheuristic feature optimization challenges	Use of deer hunting (feature) optimization followed by GRU RNN
Hameed et al. [29]	IoT	Logistic regression + MLP ANN	Supervised learning for feature selection, frequent re-training	Scheme outperforms ML methods in smart city IoT deployments
Guan et al. [30]	IoT	CNN, LSTM	Reliance on determining appropriate data transfer models from other domain(s)	High classification accuracy using transfer learning approaches from existing classifiers
Zafar et al. [33]	IoBNT	PSO, ANN	Limited data, and feature diversity	NN-based profiling of BioFET interfacing for anomaly detection
Proposed Hybrid Classifier	IoBNT	CNN, LSTM	Latency-efficiency trade-off compared to 1D CNN	High-accuracy single-stage hybrid structure, eliminating manual feature engineering

## Data Availability

The data presented in this study are openly available in Zenodo at doi:10.5281/zenodo.6640948, reference number [52].

## References

[1] Akyildiz I.F., Pierobon M., Balasubramaniam S., Koucheryavy Y. (2015). The internet of Bio-Nano things. IEEE Commun. Mag..

[2] Nakano T., Kobayashi S., Suda T., Okaie Y., Hiraoka Y., Haraguchi T. (2014). Externally Controllable Molecular Communication. IEEE J. Sel. Areas Commun..

[3] Akyildiz I.F., Brunetti F., Blázquez C. (2008). Nanonetworks: A new communication paradigm. Comput. Netw..

[4] Zafar S., Nazir M., Bakhshi T., Khattak H.A., Khan S., Bilal M., Choo K.-K.R., Kwak K.-S., Sabah A. (2021). A Systematic Review of Bio-Cyber Interface Technologies and Security Issues for Internet of Bio-Nano Things. IEEE Access.

[5] Chude-Okonkwo U.A.K., Malekian R., Maharaj B.T. (2016). Biologically Inspired Bio-Cyber Interface Architecture and Model for Internet of Bio-NanoThings Applications. IEEE Trans. Commun..

[6] Nakano T., Moore M.J., Wei F., Vasilakos A.V., Shuai J. (2012). Molecular Communication and Networking: Opportunities and Challenges. IEEE Trans. NanoBiosci..

[7] Nakano T., Okaie Y., Vasilakos A.V. (2013). Transmission Rate Control for Molecular Communication among Biological Nanomachines. IEEE J. Sel. Areas Commun..

[8] Felicetti L., Femminella M., Reali G., Nakano T., Vasilakos A.V. (2014). TCP-Like Molecular Communications. IEEE J. Sel. Areas Commun..

[9] Garralda N., Llatser I., Cabellos-Aparicio A., Alarcón E., Pierobon M. (2011). Diffusion-based physical channel identification in molecular nanonetworks. Nano Commun. Netw..

[10] Kuran M., Yilmaz H.B., Tugcu T., Özerman B. (2010). Energy model for communication via diffusion in nanonetworks. Nano Commun. Netw..

[11] Gregori M., Akyildiz I.F. (2010). A new nanonetwork architecture using flagellated bacteria and catalytic nanomotors. IEEE J. Sel. Areas Commun..

[12] Deshpande P.P., Biswas S., Torchilin V.P. (2013). Current trends in the use of liposomes for tumor targeting. Nanomedicine.

[13] Torchilin V.P. (2014). Multifunctional, stimuli-sensitive nanoparticulate systems for drug delivery. Nat. Rev. Drug Discov..

[14] Dollard M.-A., Billard P. (2003). Whole-cell bacterial sensors for the monitoring of phosphate bioavailability. J. Microbiol. Methods.

[15] Yeo D., Wiraja C., Chuah Y.J., Gao Y., Xu C. (2015). A nanoparticle-based sensor platform for cell tracking and status/function assessment. Sci. Rep..

[16] Lee Y.-E.K., Smith R., Kopelman R. (2009). Nanoparticle PEBBLE sensors in live cells and in vivo. Annu. Rev. Anal. Chem..

[17] Kuscu M., Akan O.B. (2016). Modeling and Analysis of SiNW FET-Based Molecular Communication Receiver. IEEE Trans. Commun..

[18] Liu Y., Tsao C., Kim E., Tschirhart T., Terrell J.L., Bentley W.E., Payne G.F. (2017). Using a Redox Modality to Connect Synthetic Biology to Electronics: Hydrogel-Based Chemo-Electro Signal Transduction for Molecular Communication. Adv. Healthc. Mater..

[19] Liu Y., Li J., Tschirhart T., Terrell J.L., Kim E., Tsao C., Kelly D.L., Bentley W.E., Payne G.F. (2017). Connecting Biology to Electronics: Molecular Communication via Redox Modality. Adv. Healthc. Mater..

[20] Dressler F., Kargl F. Security in nano communication: Challenges and open research issues. Proceedings of the ICC 2012–2012 IEEE International Conference on Communications.

[21] Loscri V., Marchal C., Mitton N., Fortino G., Vasilakos A.V. (2014). Security and Privacy in Molecular Communication and Networking: Opportunities and Challenges. IEEE Trans. NanoBiosci..

[22] Giaretta A., Balasubramaniam S., Conti M. (2016). Security Vulnerabilities and Countermeasures for Target Localization in Bio-NanoThings Communication Networks. IEEE Trans. Inf. Forensics Secur..

[23] Zafar S., Aman W., Rahman M.M.U., Alomainy A., Abbasi Q.H. Channel impulse response-based physical layer authentication in a diffusion-based molecular communication system. Proceedings of the 2019 UK/China Emerging Technologies (UCET).

[24] El-Fatyany A., Wang H., El-Atty S.M.A., Khan M. (2020). Biocyber Interface-Based Privacy for Internet of Bio-nano Things. Wirel. Pers. Commun..

[25] Bakhshi T., Shahid S. Securing internet of bio-nano things: ML-enabled parameter profiling of bio-cyber interfaces. Proceedings of the 2019 22nd International Multitopic Conference (INMIC).

[26] Abusitta A., de Carvalho G.H., Wahab O.A., Halabi T., Fung B.C., Al Mamoori S. (2023). Deep learning-enabled anomaly detection for IoT systems. Internet Things.

[27] Rosero-Montalvo P.D., István Z., Tözün P., Hernandez W. (2023). Hybrid Anomaly Detection Model on Trusted IoT Devices. IEEE Internet Things J..

[28] Chander N., Kumar M.U. (2023). Metaheuristic feature selection with deep learning enabled cascaded recurrent neural network for anomaly detection in Industrial Internet of Things environment. Clust. Comput..

[29] Hameed A., Violos J., Leivadeas A. (2022). A Deep Learning Approach for IoT Traffic Multi-Classification in a Smart-City Scenario. IEEE Access.

[30] Guan J., Cai J., Bai H., You I. (2021). Deep transfer learning-based network traffic classification for scarce dataset in 5G IoT systems. Int. J. Mach. Learn. Cybern..

[31] Etemadi A., Farahnak-Ghazani M., Arjmandi H., Mirmohseni M., Nasiri-Kenari M. (2023). Abnormality Detection and Localization Schemes Using Molecular Communication Systems: A Survey. IEEE Access.

[32] Janabi A.H., Kanakis T., Johnson M. (2022). Convolutional Neural Network Based Algorithm for Early Warning Proactive System Security in Software Defined Networks. IEEE Access.

[33] Zafar S., Nazir M., Sabah A., Jurcut A.D. (2021). Securing Bio-Cyber Interface for the Internet of Bio-Nano Things using Particle Swarm Optimization and Artificial Neural Networks based parameter profiling. Comput. Biol. Med..

[34] Lopez-Martin M., Carro B., Sanchez-Esguevillas A., Lloret J. (2017). Network Traffic Classifier With Convolutional and Recurrent Neural Networks for Internet of Things. IEEE Access.

[35] Bakhshi T., Ghita B. (2021). Anomaly Detection in Encrypted Internet Traffic Using Hybrid Deep Learning. Secur. Commun. Netw..

[36] Li X., Zhang D., Li M., Lee D.-J. (2022). Accurate Head Pose Estimation Using Image Rectification and a Lightweight Convolutional Neural Network. IEEE Trans. Multimed..

[37] Goodwin I., Bengio Y., Courville A. (2016). Deep Learning.

[38] Abiodun O.I., Jantan A., Omolara A.E., Dada K.V., Mohamed N.A., Arshad H. (2018). State-of-the-art in artificial neural network applications: A survey. Heliyon.

[39] Tealab A. (2018). Time series forecasting using artificial neural networks methodologies: A systematic review. Future Comput. Inform. J..

[40] Olvera D., Monaghan M.G. (2021). Electroactive material-based biosensors for detection and drug delivery. Adv. Drug Deliv. Rev..

[41] Nakano T., Suda T., Okaie Y., Moore M.J., Vasilakos A.V. (2014). Molecular communication among biological nanomachines: A layered architecture and research issues. IEEE Trans. NanoBiosci..

[42] Klein T., Bradley G. (2019). Cunningham’s Textbook of Veterinary Physiology.

[43] Marques S.M., da Silva J.C.G.E. (2009). Firefly bioluminescence: A mechanistic approach of luciferase catalyzed reactions. IUBMB Life.

[44] Bakhshi T. (2022). Hybrid Deep Learning Techniques for Securing Bioluminescent Interfaces in Internet of Bio Nano Things (1.1) [Data set]. Zenodo.

[45] Zeng Y., Gu H., Wei W., Guo Y. (2019). $Deep-Full-Range$: A Deep Learning Based Network Encrypted Traffic Classification and Intrusion Detection Framework. IEEE Access.

[46] Chollet F., Keras (2015). https://keras.io.

[47] Dixit P., Silakari S. (2021). Deep Learning Algorithms for Cybersecurity Applications: A Technological and Status Review. Comput. Sci. Rev..

[48] Google TensorFlow. https://www.tensorflow.org.

[49] Springenberg J.T., Dosovitskiy A., Brox T., Riedmiller M. (2014). Striving for Simplicity: The All-Convolutional Net. arXiv.

[50] Burr G.W., Shelby R.M., Sebastian A., Kim S., Kim S., Sidler S., Virwani K., Ishii M., Narayanan P., Fumarola A. (2017). Neuromorphic computing using non-volatile memory. Adv. Physics X.

[51] Intel Loihi 2: A New Generation of Neuromorphic Computing. https://www.intel.com/content/www/us/en/research/neuromorphic-computing.html.

[52] Esser S.K., Merolla P.A., Arthur J.V., Cassidy A.S., Appuswamy R., Andreopoulos A., Berg D.J., McKinstry J.L., Melano T., Barch D.R. (2016). Convolutional networks for fast, energy-efficient neuromorphic computing. Proc. Natl. Acad. Sci. USA.

[53] Boybat I., Le Gallo M., Nandakumar S.R., Moraitis T., Parnell T., Tuma T., Rajendran B., Leblebici Y., Sebastian A., Eleftheriou E. (2018). Neuromorphic computing with multi-memristive synapses. Nat. Commun..

[54] Davison A.P., Brüderle D., Eppler J., Kremkow J., Muller E., Pecevski D., Perrinet L., Yger P. (2009). PyNN: A common interface for neuronal network simulators. Front. Neurosci..

[55] Wunderlich T., Kungl A.F., Müller E., Hartel A., Stradmann Y., Aamir S.A., Grübl A., Heimbrecht A., Schreiber K., Stöckel D. (2019). Demonstrating Advantages of Neuromorphic Computation: A Pilot Study. Front. Neurosci..

[56] Li Y., Ang K.-W. (2021). Hardware Implementation of Neuromorphic Computing Using Large-Scale Memristor Crossbar Arrays. Adv. Intell. Syst..

[57] Wang G., Ma S., Wu Y., Pei J., Zhao R., Shi L. (2021). End-to-End Implementation of Various Hybrid Neural Networks on a Cross-Paradigm Neuromorphic Chip. Front. Neurosci..

[58] Wang Z., Zhao W., Kang W., Zhang Y., Klein J.-O., Chappert C. Ferroelectric tunnel memristor-based neuromorphic network with 1T1R crossbar architecture. Proceedings of the 2014 International Joint Conference on Neural Networks (IJCNN).

